# Handgrip strength and the prognosis of patients with heart failure: A meta‐analysis

**DOI:** 10.1002/clc.24063

**Published:** 2023-07-19

**Authors:** Yu Wang, Xuehua Pu, Zhiyun Zhu, Wenbin Sun, Lu Xue, Jilu Ye

**Affiliations:** ^1^ Department of Critical Care Medicine Taizhou People's Hospital Taizhou Jiangsu Province China

**Keywords:** frailty, handgrip strength, heart failure, meta‐analysis, mortality

## Abstract

**Background:**

Reduced muscular strength is common in patients with heart failure (HF). The aim of the systematic review and meta‐analysis was to evaluate the association between handgrip strength (HGS) and prognosis of patients with HF.

**Hypothesis:**

Reduced HGS may be a risk factor of poor prognosis of patients with HF.

**Methods:**

Relevant observational studies with longitudinal follow‐up were obtained by a comprehensive search of PubMed, Embase, Cochrane Library, and Web of Science databases. A random‐effects model was used to pool the results.

**Results:**

Fifteen studies involving 7350 patients with HF were included in the meta‐analysis. Pooled results showed that HF patients with lower HGS were associated with a higher risk of mortality during follow‐up (risk ratio [RR]: 2.00, 95% confidence interval [CI]: 1.55–2.58, *p* < .001; *I* ^2^ = 0%). Subgroup analysis showed that the association was not significantly affected by characteristics such as study country, design, mean age of the patients, HF status (stable or advanced/acute), HF type (reduced or preserved ejection fraction), follow‐up duration, and quality score (*p* for subgroup difference all > 0.05). Further analysis showed that per 1 kgf decrease of HGS was associated with an 8% increased risk of mortality during follow‐up (RR: 1.08, 95% CI: 1.05–1.11, *p* < .001; *I* ^2^ = 12%). Moreover, HF patients with lower HGS were also related to a higher risk of composite outcome of HF rehospitalization or mortality (RR: 1.67, 95% CI: 1.19–2.35, *p* = .003; *I* ^2^ = 53%).

**Conclusion:**

A low HGS may be associated with poor clinical outcomes of patients with HF.

## Graphical abstract

A meta‐analysis of 15 studies showed that low handgrip strength may be associated with poor clinical outcomes, such as all‐cause mortality and a composite outcome of rehospitalization and mortality of patients with heart failure.

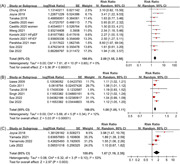

## 1 BACKGROUND

Globally, approximately 26 million people are suffering from heart failure (HF), the end stage of various cardiovascular diseases (CVDs).^1^, ^2^ Over the next few decades, the improved treatment methods for CVDs and the accelerated aging of the global population will drive the number of people suffering from HF to grow continually.^1^ Pathophysiologically, HF is characterized by initial cardiac dysfunction caused by various etiologies and subsequent activation of the systemic neuro‐hormonal system and inflammation.^3^, ^4^ During the progression of HF, inflammation plays multiple roles which not only include myocardial remodeling but also reduction of peripheral muscular strength and mass.^5^ As an indicator of sarcopenia and physical frailty, reduced muscular strength is shown to be prevalent in patients with HF, particularly in the older patients.^6^ Clinically, it has been proven that handgrip strength (HGS) is a reliable indicator of muscle strength in adults.^7^ The HGS is a simple, inexpensive, and easily measured muscle strength metric using a muscle strength dynamometer, which makes it one of the most commonly used indicators of muscle strength.^8^ Accumulating studies indicate that a low HGS may be a marker of increased mortality risk in older people.^9^, ^10^ Accumulating studies suggest that besides causing low physical performance and reduced cardiorespiratory fitness in older HF patients, reduced HGS in HF may also be a prognostic factor for poor survival.^11^, ^12^ However, previous studies evaluating the prognostic role of HGS in patients with HF retrieved inconsistent results.^13^, ^14^, ^15^, ^16^, ^17^, ^18^, ^19^, ^20^, ^21^, ^22^, ^23^, ^24^, ^25^, ^26^, ^27^ Therefore, the aim of the study was to evaluate the association between HGS and prognosis of patients with HF by conducting a systematic review and meta‐analysis.

## 2 METHODS

The meta‐analysis was performed in accordance with the MOOSE (Meta‐analysis of Observational Studies in Epidemiology).^28^

### 2.1 Literature search

Studies were identified via systematic search of electronic databases including PubMed, Embase, Cochrane library, and Web of Science from inception to December 17, 2022. Cochrane Library was searched because the post hoc analyses of clinical trials which fit the aim of the meta‐analysis could also be included. A combined search strategy was used, which included: (1) “handgrip” OR “hand strength” OR “muscle strength dynamometer” and (2) “heart failure” OR “cardiac failure” OR “cardiac dysfunction”. The search was limited to clinical studies published in English. The reference lists of related original and review articles were also analyzed using a manual approach.

### 2.2 Study selection

The inclusion criteria for the studies were: (1) studies that are designed to follow‐up over time, such as cohort studies, post hoc analyses of clinical trials, or nested case–control studies; (2) included patients with confirmed diagnosis of HF at baseline; (3) HGS was measured with a muscle strength dynamometer and considered as the exposure; (4) patients were followed for at least 3 months and reported the incidence of all‐cause mortality and/or the composite outcome of HF‐rehospitalization or mortality during follow‐up; and (5) the associations between HGS and the above outcomes were reported as risk ratio (RR) and corresponding 95% confidence interval [CI], or these data could be calculated or estimated. Studies with HGS analyzed as continuous or categorized variables were both included. The cutoffs for defining low versus normal HGS were in accordance with the criteria used in the original studies. The minimal follow‐up durations were limited to 3 months because we did not want to investigate the acute relationship between HGS and clinical outcomes of HF. Only full‐length articles published in peer‐reviewed journals were included. Gray literatures (conference abstracts or unpublished data) were not considered because these literatures were generally not peer reviewed. Reviews, editorials, cross‐sectional studies, studies including patients without HF, studies not measuring HGS, or studied not reporting the outcomes of interest were excluded.

### 2.3 Data extracting and quality evaluation

Literature search, data extraction, and quality assessment of the included studies were independently performed by two authors according to the predefined criteria. Discrepancies were resolved by consensus between the two authors. The extracted data included: (1) general information of the study (author, year, and country); (2) study design characteristics; (3) patient and disease characteristics, such as diagnosis, age, sex, disease status (stable/chronic or advanced/acute), left ventricular ejection fraction (LVEF), and the New York Heart Association (NYHA) class; (4) exposure characteristics (analyzing methods and cutoffs for HGS); (5) follow‐up durations and outcomes reported; and (6) potential confounding factors adjusted in the multivariate regression analysis model for the association between HGS and prognosis of patients with HF. The quality of each study was evaluated using the Newcastle‐Ottawa Scale (NOS)^29^ which ranges from 1 to 9 stars and judges each study regarding three aspects: selection of the study groups; the comparability of the groups; and the ascertainment of the outcome of interest.

### 2.4 Statistical analyses

We used RR and the corresponding 95% CI as the general measure for association between HGS and the clinical outcomes of patients with HF during follow‐up. For study that reported hazard ratio (HR), HR was directly considered as RR, while for studies that reported odds ratio (OR), data were converted to RR for the meta‐analysis as previously reported (RR = OR/([1 − pRef] + [pRef × OR]), where pRef is the prevalence of the outcome in the reference group (normal HGS group).^30^ For studies with HGS analyzed as categorized variables, RRs for the incidence of mortality or the composite outcome between HF patients with decreased versus normal HGS were calculated. For studies with HGS analyzed as the continuous variables, RRs for incidence of the above outcomes with 1 kgf decrease of HGS were estimated. Data of RR and the corresponding stand error (SE) were calculated from 95% CI or *p* values, and were logarithmically transformed to stabilize the variance and normalize the distribution.^31^ The Cochrane *Q* test was used to evaluate the heterogeneity among the included studies.^32^ Besides, the *I* ^2^ statistic was also estimated.^32^ A significant heterogeneity was considered if *I* ^2^ > 50%. We used a random‐effect model to synthesize the RR data because this model is considered as a more generalized method which incorporates the potential heterogeneity among the included studies.^33^ Influencing analysis by sequentially excluding one data set at a time was performed to evaluate the stability of the findings. Influencing analysis is also called sensitivity analysis, aiming to determine if the overall result of the meta‐analysis is primarily driven by either of the included data set. If yes, the results were considered to be unstable.^31^ For meta‐analysis with at least 10 data sets, subgroup analyses were also performed to investigate the influences of study characteristics on the association, such as study country, design, mean age of the patients, HF status (stable or advanced/acute), HF type (reduced or preserved ejection fraction), analytic model (multivariate or univariate), follow‐up duration, and quality score. The potential publication bias was assessed by funnel plots. The Egger's regression asymmetry test^34^ was also performed if at least 10 data sets were included. A *p* value <.05 indicates statistically significance. We used the RevMan (Version 5.1; Cochrane Collaboration) and Stata 12.0 software for the meta‐analysis and statistical analysis.

## 3 RESULTS

### 3.1 Literature search

**Figure 1:**
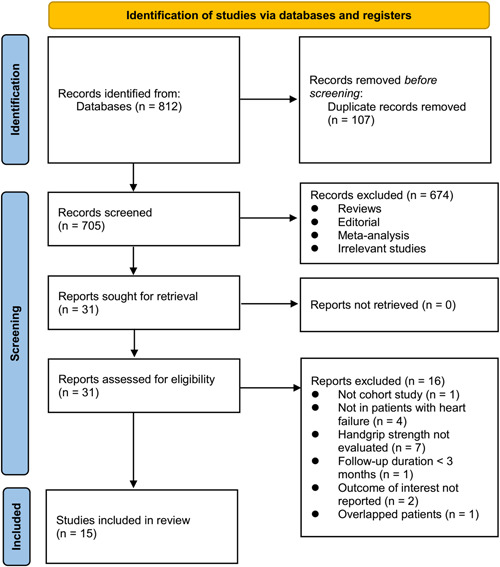
Diagram of database search and study identification.

The process of database search is summarized in Figure 1. Briefly, 812 articles were found via initial literature search, and 705 articles were left after excluding the duplicated records. Subsequently, 674 articles were excluded by screening the titles and abstracts because they were not relevant to the objective of the meta‐analysis. Accordingly, 31 articles underwent full‐text review, and 16 of them were further excluded for the reasons listed in Figure 1. Finally, 15 studies were included in the meta‐analysis.^13^, ^14^, ^15^, ^16^, ^17^, ^18^, ^19^, ^20^, ^21^, ^22^, ^23^, ^24^, ^25^, ^26^, ^27^

### 3.2 Study characteristics and quality evaluation

The characteristics of the included studies are summarized in Table 1. Overall, 15 cohort studies involving 7350 patients with HF were included in the meta‐analysis.^13^, ^14^, ^15^, ^16^, ^17^, ^18^, ^19^, ^20^, ^21^, ^22^, ^23^, ^24^, ^25^, ^26^, ^27^ These studies were published between 2009 and 2022, and performed in Japan, Mexico, the United States, China, India, Brazil, the United Kingdom, and Singapore. Regarding study design, nine of them were prospective cohort studies,^13^, ^19^, ^20^, ^21^, ^23^, ^24^, ^25^, ^26^, ^27^ and the other six were retrospective cohort studies.^14^, ^15^, ^16^, ^17^, ^18^, ^22^ As for the diagnosis of the patients, eight studies included patients with stable or chronic HF,^13^, ^14^, ^18^, ^19^, ^21^, ^22^, ^26^, ^27^ while the other seven include patients with advanced or acute HF.^15^, ^16^, ^17^, ^20^, ^23^, ^24^, ^25^ The sample size of the included studies varied from 56 to 1778. The mean ages of the patients varied from 60 to 83 years, and the proportions of men ranged from 53% to 100%. The proportions of patients with HF with reduced ejection fraction (HFrEF) and ischemic HF also ranged among the included studies. For all of the included studies, HGS of the patients was measured with a muscle strength dynamometer. The HGS was analyzed as a continuous variable in six studies,^13^, ^14^, ^17^, ^21^, ^24^, ^26^ and as a categorized variable in 12 studies.^15^, ^16^, ^17^, ^18^, ^19^, ^20^, ^22^, ^23^, ^24^, ^25^, ^26^, ^27^ The follow‐up durations varied from 3 to 43 months, and the outcomes of all‐cause mortality and composite outcome of HF rehospitalization and mortality were reported in 13^13^, ^14^, ^15^, ^16^, ^17^, ^18^, ^19^, ^20^, ^21^, ^22^, ^24^, ^26^, ^27^ and 4 studies.^16^, ^23^, ^25^, ^27^ When the relationships between HGS and the clinical outcomes of patients with HF were estimated, univariate regression analyses were used in 3 studies,^15^, ^16^, ^20^ while in the other 12 studies, multivariate regression analyses were used,^13^, ^14^, ^17^, ^18^, ^19^, ^21^, ^22^, ^23^, ^24^, ^25^, ^26^, ^27^ which incorporated the potential confounding factors including age, sex, body mass index, left ventricular ejection fraction (LVEF), and comorbidities, and so forth. The NOS of the included studies was between 5 and 9 stars, indicating moderate to good study quality (Table 2).

**Table 1:** Characteristics of the included cohort studies.

Reference	Country	Design	Diagnosis	Sample size	Mean age (years)	Male (%)	HFrEF (%)	Ischemic (%)	NYHA class	LVEF (%)	HGS analysis and cutoff	Follow‐up duration (months)	Outcomes	Variables adjusted
Izawa et al.^13^	Japan	PC	Outpatients with stable CHF	148	62.8	100	100	26.3	I–III	35	Continuous (per 1 kgf)	43.2	All‐cause death	Age, LVEF, and peak VO2
Colín et al.^14^	Mexico	RC	Outpatients with stable CHF	405	61.7	53.8	43.2	48.4	I–III	43.8	Continuous (per 1 kgf)	36	All‐cause death	Age, sex, LVEF, BMI, HF type, etiology, comorbidities, ARB use, and HGB
Chung et al.^15^	USA	RC	Patients with advanced HF for VAD implantation	72	59	89	100	46	II–IV	18	Categorized: 25% of BW	18	All‐cause death	None
Joyce et al.^16^	USA	RC	Older patients with acute HF at discharge	56	77	73	68	35.7	I–IV	NR	Categorized, gender and BMI derived cutoff by the Fried criteria	6	All‐cause death, composite outcome	None
Tanaka et al.^17^	Japan	RC	Older patients admitted for acute HF	603	74.9	62.7	29.9	24.4	II–IV	48.2	Continuous (per 1 kgf) and categorized (Q4:Q1)	20.4	All‐cause death	Age, sex, MAGGIC risk score, comorbidities, and BNP
Castillo et al.^18^	Mexico	RC	Patients with stable CHF	546	60.8	53.3	NR	58.1	I–IV	42.8	Categorized: men < 10.1 kg/m^2^, women < 7.95 kg/m^2^	36	All‐cause death	Age, sex, LVEF, BMI, NYHA class, and fluid alteration
Weng et al.^22^	China	RC	Older patients with CHF	151	82.7	73.5	42.4	57	I–IV	52	Categorized: T3:T1–2	38.4	All‐cause death	Age, sex, severity of HF, CCI, laboratory tests, and medication
Konishi et al.^19^	Japan	PC	Older patients with stable CHF	942	79	58.4	49.6	34	I–IV	46	Categorized: men < 26 kgf, women < 18 kgf	12	All‐cause death	Age, sex, malignancy, severe AR, BNP, the MAGGIC risk score, and each of sarcopenia or low muscle mass
Yamada et al.^23^	Japan	PC	Older patients with acute HF at discharge	1778	76	60.5	38.4	26.4	I–IV	44	Categorized: men < 30 kgf, women < 17.5 kgf	24	Composite outcome	Age, sex, BMI LVEF, history of HF, comorbidities, biochemical data, medications, cognitive function, and depression
Singh et al.^21^	India	PC	Outpatients with stable CHF	210	60.6	61.7	100	44.5	I–V	30.2	Continuous (per 1 kgf)	12	All‐cause death	Age, sex, BMI LVEF, HGB, and BNP
Parahiba et al.^20^	Brazil	PC	Patients admitted for acute HF	161	68	62	NR	34	III–IV	37.7	Categorized: men 25.5 kgf	3	All‐cause death	None
Sze et al.^26^	UK	PC	Outpatients with stable CHF	467	76	67	62	42	I–IV	45	Continuous (per 1 kgf) and categorized: gender and BMI derived cutoff by the Fried criteria	18.5	All‐cause death	Age, sex, BMI, AF, NYHA class, CCI, BNP, HGB, serum sodium, and eGFR
Yamamoto et al.^27^	Japan	PC	Older patients with stable CHF	1215	81	57.2	50.9	35.9	I–IV	45	Categorized: men < 26 kgf, women < 18 kgf	12	All‐cause death and composite outcome	Age, sex, BMI, eGFR, NYHA class, comorbidities, albumin, HGB, sodium level, BNP, serum sodium, eGFR, and CV medications
Lala et al.^25^	USA	PC	Patients with advanced HF	345	60	75	100	NR	I–IV	20	Categorized, gender and BMI derived cutoff by the Fried criteria	12	Composite outcome	Age, sex, BMI, albumin, Scr, and LVEF
Dai et al.^24^	Singapore	PC	Patients admitted for acute HF	251	66.5	71.7	NR	NR	III–IV	NR	Continuous (per 1 kgf) and categorized (<5% percentile)	36	All‐cause death	Age, sex, comorbidities, and total FACT‐G score

Abbreviations: AF, atrial fibrillation; AR, aortic regurgitation; ARB, angiotensin receptor II blocker; BMI, body mass index; BNP, B‐natriuretic peptide; CCI, Charlson Comorbidity Index; CHF, chronic heart failure; CV, cardiovascular; eGFR, estimated glomerular infiltrating rate; FACT‐G, The Functional Assessment of Cancer Therapy‐General; HF, heart failure; HFrEF, heart failure with reduced ejection fraction; HGB, hemoglobin; HGS, handgrip strength; LVEF, left ventricular ejection fraction; MAGGIC, the Meta‐Analysis Global Group in Chronic Heart Failure; NR, not reported; NYHA, New York Heart Association; PC, prospective cohort; Q, quartile; RC, retrospective cohort; T, tertile; VAD, ventricular‐assisting device; VO2, oxygen consumption.

**Table 2:** Study quality evaluation via the Newcastle‐Ottawa Scale.

Reference	Representativeness of the exposed cohort	Selection of the nonexposed cohort	Ascertainment of exposure	Outcome not present at baseline	Control for age and sex	Control for other confounding factors	Assessment of outcome	Enough long follow‐up duration	Adequacy of follow‐up of cohorts	Total
Izawa et al.^13^	1	1	1	1	1	1	1	1	1	9
Colín et al.^14^	0	1	1	1	1	1	1	1	1	8
Chung et al.^15^	0	1	1	1	0	0	1	1	1	6
Joyce et al.^16^	0	1	1	1	0	0	1	0	1	5
Tanaka et al.^17^	0	1	1	1	1	1	1	1	1	8
Castillo et al.^18^	0	1	1	1	1	1	1	1	1	8
Weng et al.^22^	0	1	1	1	1	1	1	1	1	8
Konishi et al.^19^	1	1	1	1	1	1	1	1	1	9
Yamada et al.^23^	1	1	1	1	1	1	1	1	1	9
Singh et al.^21^	1	1	1	1	1	1	1	1	1	9
Parahiba 2021^20^	1	1	1	1	0	0	1	0	1	6
Sze et al.^26^	1	1	1	1	1	1	1	1	1	9
Yamamoto et al.^27^	1	1	1	1	1	1	1	1	1	9
Lala et al.^25^	1	1	1	1	1	1	1	1	1	9
Dai et al.^24^	1	1	1	1	1	1	1	1	1	9

### 3.3 HGS and mortality of patients with HF

**Figure 2:**
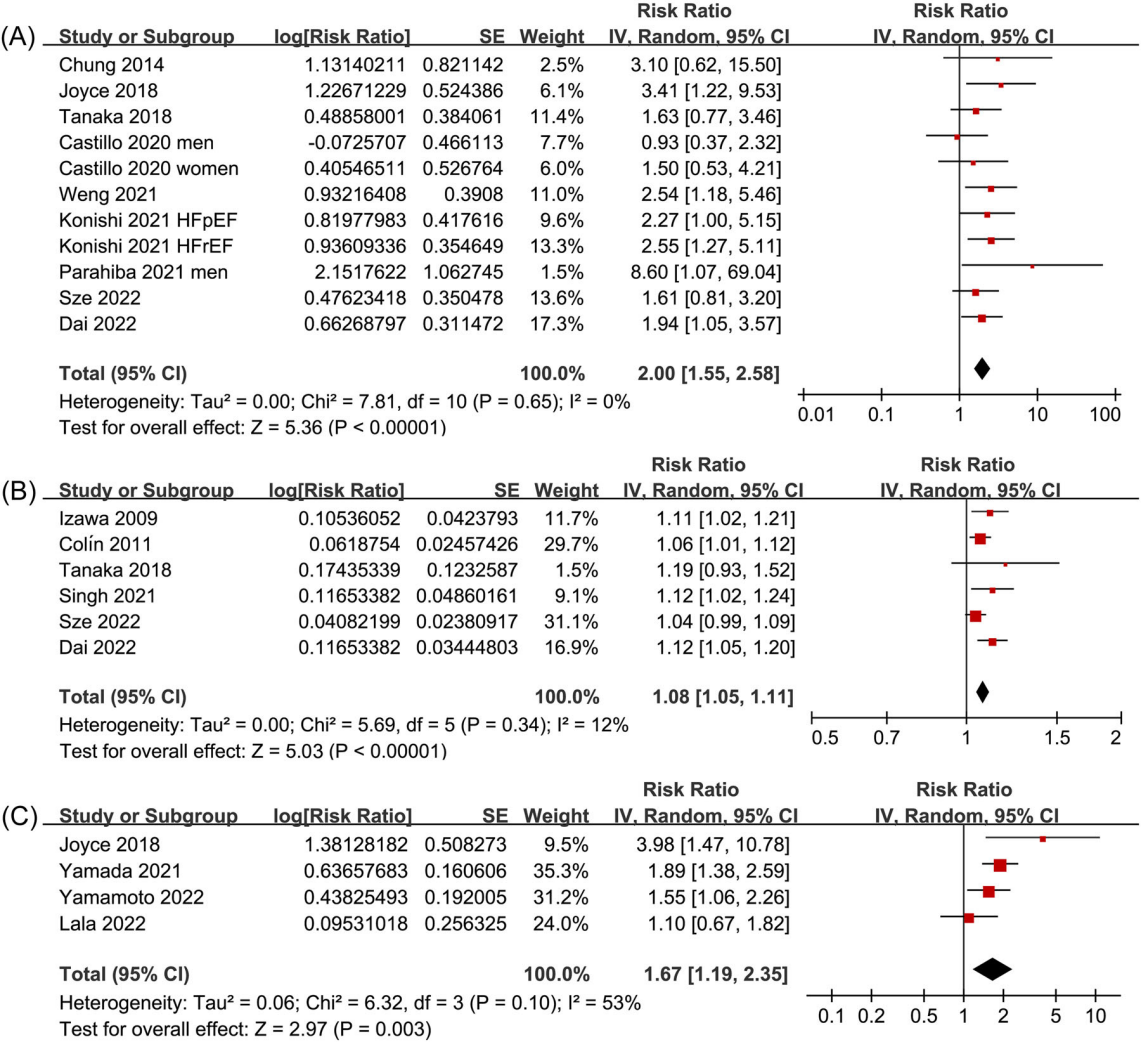
Forest plots for the meta‐analysis of the association between handgrip strength (HGS) and the prognosis of patients with heart failure (HF). (A) Forest plots for the association between HGS as a categorized variable and the mortality of patients with HF. (B) Forest plots for the association between HGS as a continuous variable and the mortality of patients with HF. (C) Forest plots for the association between HGS as a categorized variable and the composite outcome of HF rehospitalization or mortality of patients with HF.

Nine studies reported the association between HGS defined as categorized variable and the risk of all‐cause mortality in patients with HF.^15^, ^16^, ^17^, ^18^, ^19^, ^20^, ^22^, ^24^, ^26^ Since one study reported the data sets of male and female patients separately,^18^ and the other study reported the data sets of patients with HFrEF and HFpEF separately,^19^ these data sets were included independently. Overall, 11 data sets from 9 studies were included for the meta‐analysis of HGS and all‐cause mortality. Pooled results showed that HF patients with lower HGS were associated with a higher risk of mortality during follow‐up (RR: 2.00, 95% CI: 1.55–2.58, *p* < .001; Figure 2A) with no significant heterogeneity (*p* for Cochrane *Q* test = 0.65, *I* ^2^ = 0%). Influencing analysis by excluding one data set at a time showed similar results (RR: 1.93–2.14, *p* all < 0.05; Supporting Information: Table 1). Subgroup analysis showed that the association was consistent in patients from Asian and non‐Asian countries, in prospective and retrospective cohort studies, in patients with mean ages < and ≥70 years, in studies of stable/chronic or advanced/acute HF, in patients with HFrEF and HFpEF, in studies with follow‐up duration within or longer than 18 months, in studies with univariate and multivariate analyses, and in studies with different NOS (*p* for subgroup difference all >.05; Table 3 and Supporting Information: Figure 1). Further meta‐analysis including six studies^13^, ^14^, ^17^, ^21^, ^24^, ^26^ showed that per 1 kgf decrease of HGS was associated with an 8% increased risk of mortality in patients with HF during follow‐up (RR: 1.08, 95% CI: 1.05–1.11, *p* < .001; *I* ^2^ = 12%; Figure 2B). Similarly, influencing analysis by excluding one data set at a time showed consistent results (RR: 1.07–1.09, *p* all <.05; Supporting Information: Table 1A–H).

**Table 3:** Subgroup analyses for the association between HGS and mortality in patients with HF.

Study characteristics	Data sets number	RR (95% CI)	*I* ^2^	*p* for subgroup effect	*p* for subgroup difference
Country					
Asian	5	2.14 [1.55, 2.95]	0%	<.001	
Non‐Asian	6	1.86 [1.14, 3.02]	21%	.01	.64
Design					
Prospective	5	2.12 [1.50, 2.98]	0%	<.001	
Retrospective	6	1.87 [1.28, 2.73]	0%	.001	.63
Mean age					
<70 years	5	1.75 [1.07, 2.88]	16%	.03	
≥70 years	6	2.16 [1.58, 2.96]	0%	<.001	.48
Disease status					
Stable HF	6	1.89 [1.37, 2.61]	0%	<.001	
Advanced or acute HF	5	2.20 [1.46, 3.31]	0%	<.001	.57
HF type					
HFrEF	3	2.10 [1.31, 3.34]	0%	.002	
HFpEF	1	2.27 [1.00, 5.15]	—	.05	.87
Follow‐up durations					
Within 18 months	5	2.79 [1.79, 4.34]	0%	<.001	
More than 18 months	6	1.70 [1.25, 2.32]	0%	<.001	.07
Regression model					
Multivariate	8	1.86 [1.43, 2.43]	0%	<.001	
Univariate	3	3.82 [1.72, 8.49]	0%	.001	.10
Study quality					
NOS 5–7	3	3.82 [1.72, 8.49]	0%	.001	
NOS 8–9	8	1.86 [1.43, 2.43]	0%	<.001	.10

Abbreviations: CI, confidence interval; HF, heart failure; HFrEF, heart failure with reduced ejection fraction; HFpEF, heart failure with preserved ejection fraction; HGS, handgrip strength; NOS, the Newcastle‐Ottawa Scale; PC, prospective cohort; RC, retrospective cohort; RR, risk ratio.

### 3.4 HGS and the composite outcome of HF rehospitalization and mortality

Four studies^16^, ^23^, ^25^, ^27^ reported the association between HGS defined as a categorized variable and the incidence of the composite outcome of HF rehospitalization or mortality in patients with HF. Pooled results showed that HF patients with low HGS were associated with a higher incidence of the composite outcome (RR: 1.67, 95% CI: 1.19–2.35, *p* = .003; *I* ^2^ = 53%; Figure 2C). Influencing analysis by excluding one data set at a time showed similar results (RR: 1.55–1.87, *p* all <.05; Supporting Information: Table 1).

### 3.5 Publication bias

The funnel plots for the meta‐analyses of the associations between HGS as a categorized variable or a continuous variable and mortality, and that between HGS as a categorized variable and the composite outcome of patients with HF are shown in Supporting Information: Figure 2A–C. The plots were symmetrical on visual inspection, suggesting low risk of publication bias. Egger's regression test also suggested a low‐risk publication underlying the meta‐analysis of the association between HGS as a categorized variable and mortality (*p* = .31). For the other two outcomes, Egger's regression tests were unable to perform because limited data sets were included.

## 4 DISCUSSION

In this meta‐analysis, we combined the results of 15 relevant cohort studies, and found that compared to patients with normal HGS, HF patients with a low HGS were associated with a higher mortality risk during follow‐up. The association between HGS and mortality in HF was independent of study characteristics such as study country, design, mean age, HF status (stable or advanced/acute), HF type (reduced or preserved EF), follow‐up duration, and quality score. Moreover, further meta‐analysis with HGS analyzed as a continuous variable showed that per 1 kgf decrease of HGS was related to an 8% increased mortality risk of patients with HF. Additionally, we also found that HF patients with a low HGS also had a higher incidence of the composite outcome of HF rehospitalization or mortality. Taken together, these findings suggest that a low HGS may be associated with poor clinical outcomes of patients with HF.

To the best of our knowledge, this study may be the first meta‐analysis which comprehensively evaluated the association between HGS and prognosis of patients with HF. The strengths of the meta‐analysis include the following. First, four commonly used electronic databases were extensively searched for available relevant studies, and we retrieved up‐to‐date literatures which investigated the possible prognostic role of HGS in patients with HF. Second, all of the included studies were cohort studies, which could therefore provide a longitudinal relationship between HGS decrease and poor clinical outcome in patients with HF. Third, for the outcome of mortality, HGS was analyzed as the categorized and the continuous variables, and the consistent results further indicated the reliability of the findings. Finally, multiple subgroup analyses were performed for the meta‐analysis of the association between HGS and mortality in HF, and the similar results of the meta‐analysis confirmed that the association was not significantly affected by these study characteristics, suggesting the robustness of the finding. Currently, the potential mechanisms underlying the association between reduced HGS and poor prognosis of patients with HF may be multifactorial. Previous studies showed that both sarcopenia^35^ and frailty^36^ are established predictors of poor prognosis in patients with HF. Reduced HGS as a reflection of muscle wasting in patients with HF has been involved as an important component for both sarcopenia and physical frailty, which may at least partly explain the prognostic role of HGS in these patients.^37^ Pathophysiologically, during the progression of HF, reduced cardiac output and systemic congestion lead to a reduction in food intake and exercise capacity, promote inflammatory responses, increase sympathetic excitability, lead to secretion changes of muscle‐related hormone, and finally result in muscular weakness.^38^ Among them, the ubiquitin–proteasome system (UPS) could be activated by systemic inflammation and oxidative stress in HF, and result in autophagy and apoptosis, ultimately leading to skeletal muscle wasting.^39^ Accordingly, reduced muscular strength in patients with HF is associated with reduced functional capacity (peak oxygen consumption), deteriorated symptoms of dyspnea, and increased risks of adverse events such as falls, osteoporosis, and fracture, which all increase the risk of mortality in patients with HF.^40^ From the clinical perspective, measuring HGS in patients with HF is convenient, inexpensive, and repeatable, which also support the use of HGS measuring as a potential prognostic tool for patients with HF. Moreover, clinical trials may be considered to evaluate if exercise or nutritional strategies to improve muscular strength could improve the prognosis in patients with HF.

Our study also has limitations. Firstly, cutoffs for the defining low HGS in patients with HF varied among the included studies, which may lead to heterogeneity. However, to the best of our knowledge, there is no consensus regarding the cutoff values for the diagnosis of HGS in patients with HF. Relevant studies are needed in the future. Moreover, subgroup analysis according to the LVEF status was performed in patients with HFrEF and HFpEF. Recent guidelines of HF have raised the concept of heart failure with midrange ejection fraction (HFmrEF).^41^ However, none of the included studies reported EF status accordingly because these studies were mostly started before the concept of HFmrEF was proposed. There, it remains unknown if HGS was associated with poor prognosis of patients with HFmrEF. Studies are needed in the future to address this issue. Also, the between subgroup difference for patients with HFrEF and HFpEF is not significant (*p* = .87), which indicated that the association between HGS and mortality in HF may be independent of HF type. These findings should be interpreted with caution because limited studies were included in subgroup analyses according to HF subtype. In addition, limited studies were available for the meta‐analysis of the composite outcome in patients with HF. Besides, results of some included studies failed to show a significant association between HGS and poor outcome in patients with HF, at least partly because they are of limited sample size, which may cause statistic inadequacy (95% CI including RR = 1). Results of the meta‐analysis should be validated in large‐scale prospective studies. Moreover, although subgroup analysis limited to studies with multivariate analysis revealed consistent results, we could not exclude the possibility that there may be unadjusted factors which may confound the association between low HGS and increased mortality in patients with HF. For example, a low HGS has been related to poor nutritional status and activated inflammatory response, which are both associated with poor prognosis of patients with HF.^14^, ^42^ Therefore, the association between a low HGS and poor prognosis of HF may be confounded by malnutrition and systemic inflammation. Finally, as a meta‐analysis of observational studies, a causative relationship between low HGS and poor prognosis of patients with HF could not be confirmed based on the results. It is important to determine if interventions to increase HGS could improve the clinical outcomes in patients with HF.

## 5 CONCLUSION

To sum up, results of the meta‐analysis indicate that a low HGS may be associated with poor clinical outcomes of patients with HF, suggesting the possible prognostic importance of HGS in these patients. Future studies are needed to determine the mechanisms underlying the association between low HGS and poor prognosis of HF. More importantly, clinical studies may be considered to determine if improved HGS could reduce the mortality in patients with HF.

## AUTHOR CONTRIBUTIONS

Yu Wang and Jilu Ye designed the study. Yu Wang and Xuehua Pu performed database search, literature review, study identification, and quality evaluation. Yu Wang, Zhiyun Zhu, and Wenbin Sun performed data extraction. Yu Wang, Zhiyun Zhu, Wenbin Sun, and Lu Xue performed statistical analysis and interpreted the results. Yu Wang drafted the manuscript. Jilu Ye revised the manuscript. All the authors approved the submission of the manuscript.

## CONFLICT OF INTEREST STATEMENT

The authors declare no conflict of interest.

## Supporting information

Supporting information.

Supporting information.

Supporting information.

## Data Availability

The data sets generated and/or analyzed during the current study are available from the corresponding author upon reasonable request.
