# Supplementary material for: Handgrip strength and the prognosis of patients with heart failure: A meta‐analysis
Source: Clin Cardiol. 2023 Jul 19;46(10):1173–84. doi: 10.1002/clc.24063 (PMC10577571; doi:10.1002/clc.24063)
Supplement: Supplementary file 1 — Supporting information. [file CLC-46-1173-s003.docx]

**A**
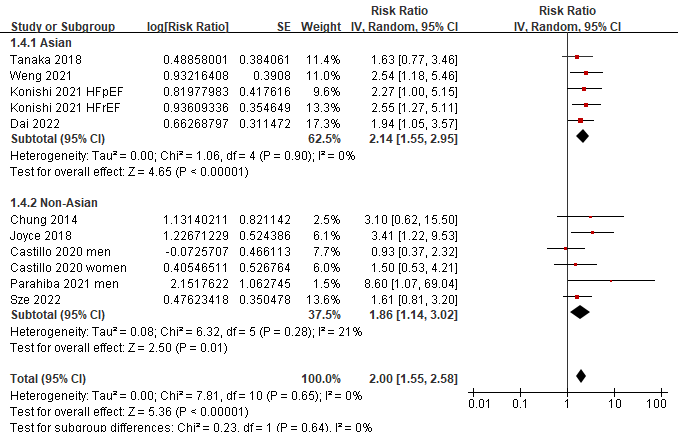


**B**
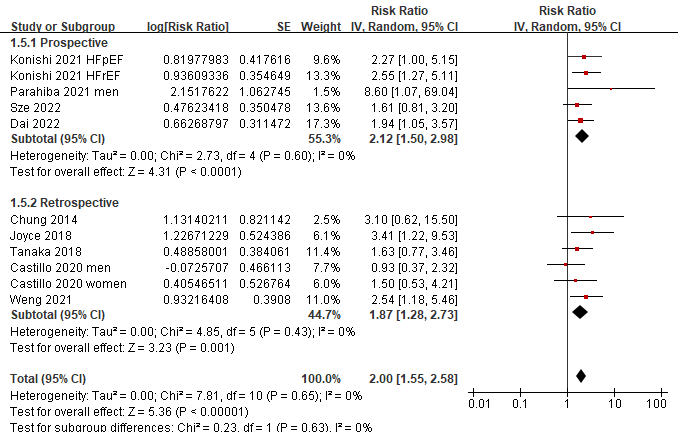


**C**
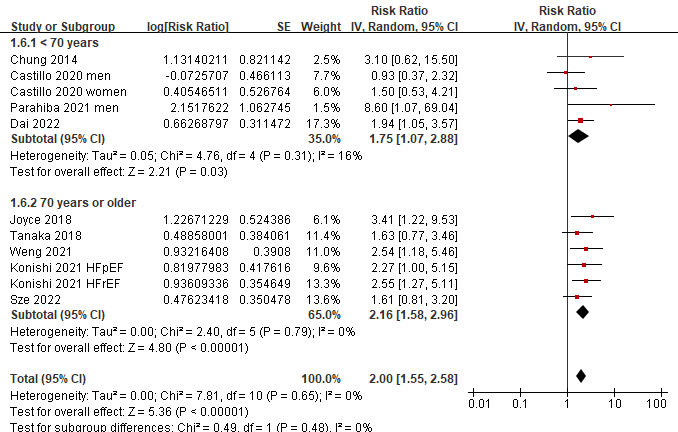


**D**
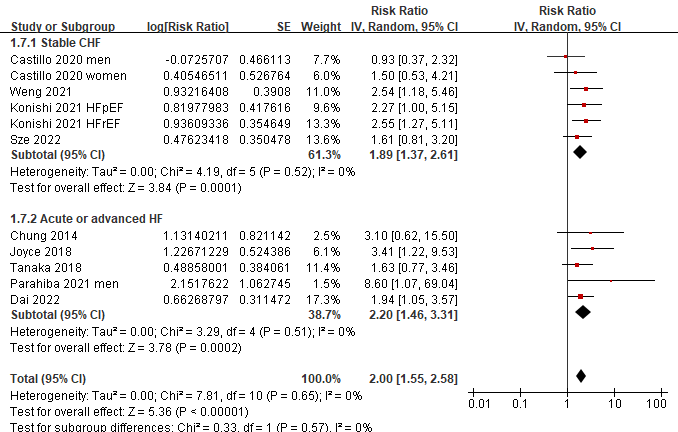


**E**
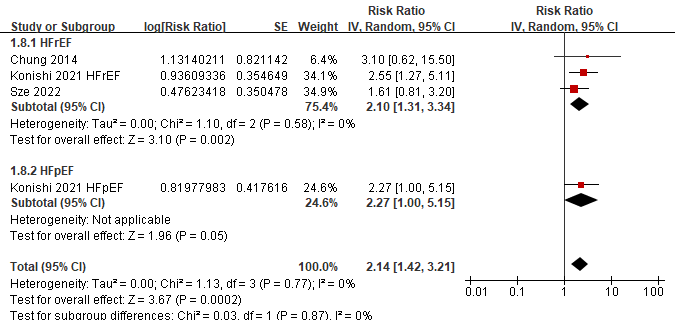


**F**
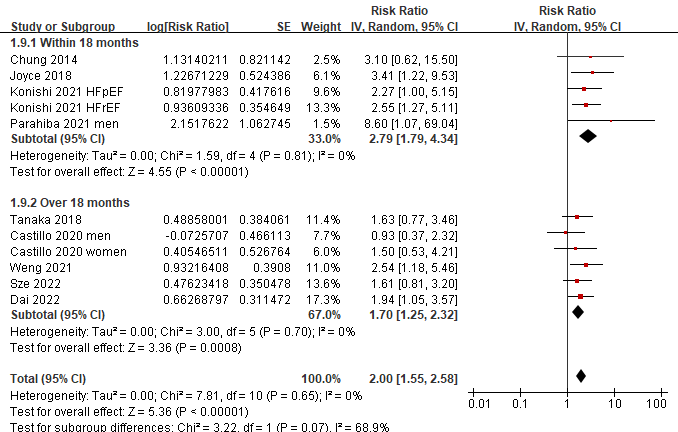


**G**
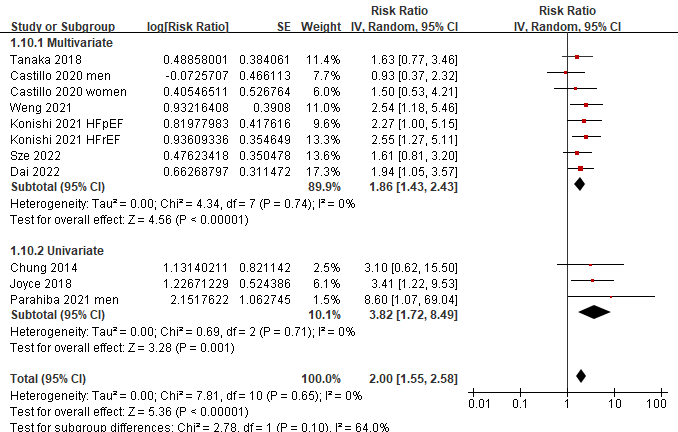


**H**
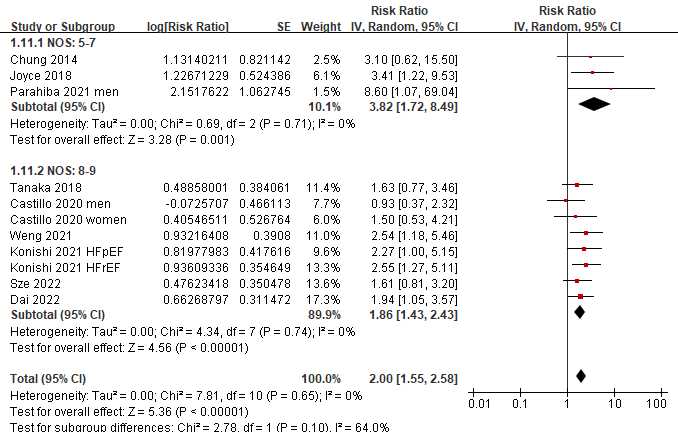


**Supplemental Figure 1** Funnel plots for the subgroup analysis of the association between HGS as categorized variable and the mortality of patients with HF; A subgroup analysis according to study country; B, subgroup analysis according to study design; C, subgroup analysis according to mean age of the patients; D, subgroup analysis according to disease status; E, subgroup analysis according to HF type; F, subgroup analysis according to follow-up durations; G, subgroup analysis according to regression model; and H, subgroup analysis according to study quality scores.
