# Supplementary material for: Handgrip strength and the prognosis of patients with heart failure: A meta‐analysis
Source: Clin Cardiol. 2023 Jul 19;46(10):1173–84. doi: 10.1002/clc.24063 (PMC10577571; doi:10.1002/clc.24063)
Supplement: Supplementary file 2 — Supporting information. [file CLC-46-1173-s001.docx]

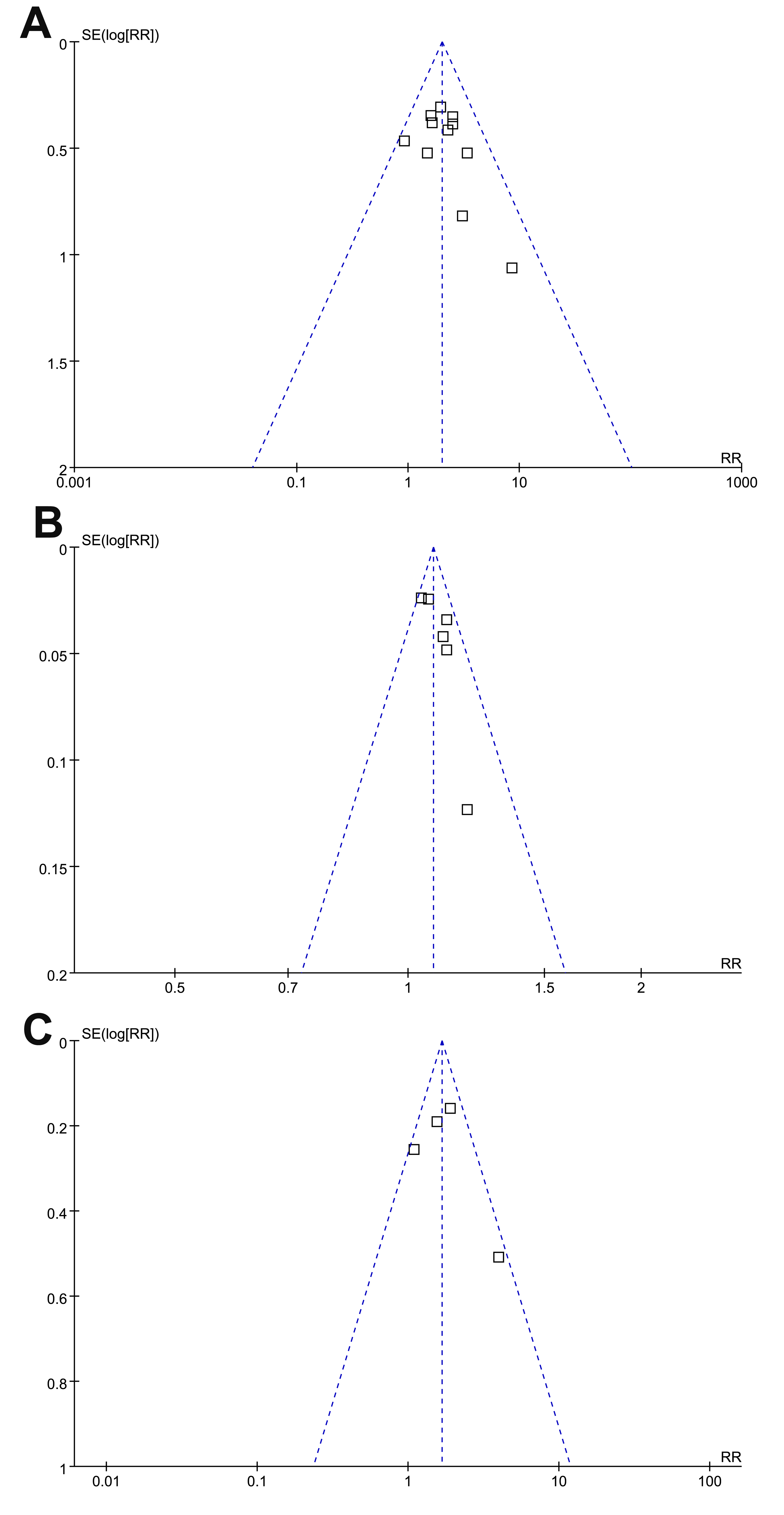


**Supplemental Figure 2** Funnel plots for the meta-analysis of the association between HGS and the prognosis of patients with HF; A, funnel plots for the association between HGS as categorized variable and the mortality of patients with HF; B, funnel plots for the association between HGS as continuous variable and the mortality of patients with HF; and C, funnel plots for the association between HGS as categorized variable and the composite outcome of HF rehospitalization or mortality of patients with HF;
