# Supplementary material for: Handgrip strength and the prognosis of patients with heart failure: A meta‐analysis
Source: Clin Cardiol. 2023 Jul 19;46(10):1173–84. doi: 10.1002/clc.24063 (PMC10577571; doi:10.1002/clc.24063)
Supplement: Supplementary file 3 — Supporting information. [file CLC-46-1173-s002.docx]

Supplemental Table 1 Results of influencing analyses

|  | RR of HGS as categorized variable for the mortality of HF | | | |
| --- | --- | --- | --- | --- |
| Dataset excluded | RR (95% CI) | P for effect | P for heterogeneity | I^2^ |
| Chung 2014 | 1.98 [1.53, 2.56] | < 0.001 | 0.58 | 0% |
| Joyce 2018 | 1.93 [1.49, 2.51] | < 0.001 | 0.67 | 0% |
| Tanaka 2018 | 2.06 [1.57, 2.69] | < 0.001 | 0.59 | 0% |
| Castillo 2020 men | 2.14 [1.64, 2.78] | < 0.001 | 0.84 | 0% |
| Castillo 2020 women | 2.04 [1.57, 2.65] | < 0.001 | 0.59 | 0% |
| Weng 2021 | 1.94 [1.49, 2.54] | < 0.001 | 0.60 | 0% |
| Konishi 2021 HFpEF | 1.98 [1.51, 2.58] | < 0.001 | 0.56 | 0% |
| Konishi 2021 HFrEF | 1.93 [1.47, 2.53] | < 0.001 | 0.61 | 0% |
| Parahiba 2021 men | 1.96 [1.52, 2.53] | < 0.001 | 0.75 | 0% |
| Sze 2022 | 2.07 [1.58, 2.72] | < 0.001 | 0.60 | 0% |
| Dai 2022 | 2.02 [1.53, 2.66] | < 0.001 | 0.60 | 0% |
|  | | | | |
|  | RR of HGS as continuous variable for the mortality of HF | | | |
| Dataset excluded | RR (95% CI) | P for effect | P for heterogeneity | I^2^ |
| Izawa 2009 | 1.08 [1.04, 1.11] | < 0.001 | 0.28 | 21% |
| Colín-Ramírez 2011 | 1.09 [1.05, 1.14] | < 0.001 | 0.25 | 25% |
| Tanaka 2018 | 1.08 [1.05, 1.11] | < 0.001 | 0.29 | 20% |
| Singh 2021 | 1.08 [1.04, 1.11] | < 0.001 | 0.30 | 18% |
| Sze 2022 | 1.09 [1.06, 1.13] | < 0.001 | 0.59 | 0% |
| Dai 2022 | 1.07 [1.04, 1.10] | < 0.001 | 0.42 | 0% |
|  | | | | |
|  | RR of HGS as categorized variable for HF rehospitalization or mortality | | | |
| Dataset excluded | RR (95% CI) | P for effect | P for heterogeneity | I^2^ |
| Joyce 2018 | 1.55 [1.17, 2.07] | 0.002 | 0.20 | 38% |
| Yamada 2021 | 1.63 [1.06, 2.51] | 0.04 | 0.08 | 61% |
| Yamamoto 2022 | 1.79 [1.04, 3.09] | 0.04 | 0.05 | 67% |
| Lala 2022 | 1.87 [1.35, 2.59] | < 0.001 | 0.21 | 36% |

HGS, handgrip strength; HF, heart failure; RR, risk ratio; CI, confidence interval;
